# Elevated levels of the methyltransferase SETD2 causes transcription and alternative splicing changes resulting in oncogenic phenotypes

**DOI:** 10.3389/fcell.2022.945668

**Published:** 2022-08-10

**Authors:** Saikat Bhattacharya, Divya Reddy, Ning Zhang, Hua Li, Jerry L. Workman

**Affiliations:** Stowers Institute for Medical Research, Kansas City, MO, United States

**Keywords:** chromatin, histone, methylation, epigenetics, cancer

## Abstract

The methyltransferase SETD2 regulates cryptic transcription, alternative splicing, and the DNA damage response. It is mutated in a variety of cancers and is believed to be a tumor suppressor. Counterintuitively, despite its important role, SETD2 is robustly degraded by the proteasome keeping its levels low. Here we show that SETD2 accumulation results in a non-canonical deposition of the functionally important H3K36me3 histone mark, which includes its reduced enrichment over gene bodies and exons. This perturbed epigenetic landscape is associated with widespread changes in transcription and alternative splicing. Strikingly, contrary to its role as a tumor suppressor, excessive SETD2 results in the upregulation of cell cycle-associated pathways. This is also reflected in phenotypes of increased cell proliferation and migration. Thus, the regulation of SETD2 levels through its proteolysis is important to maintain its appropriate function, which in turn regulates the fidelity of transcription and splicing-related processes.

## Introduction

Chromatin modifying enzymes deposit functionally important histone modifications in a spatiotemporal manner. This results in the generation of a “histone code” that can be recognized by “reader” proteins to bring about changes in vital cellular processes such as transcription, repair, and alternative splicing (Strahl and Allis, 2000; Jenuwein and Allis, 2001).

One such important histone mark is H3K36me3 which is deposited by the enzyme SETD2 in mammalian cells. SETD2’s methyltransferase activity is regulated by its interaction with RNA Pol II and the RNA-binding hnRNPs (Bhattacharya and Workman, 2020; Bhattacharya et al., 2021a; Bhattacharya et al., 2021b). Functionally, H3K36me3 correlates with transcription activation, and it also regulates DNA repair, pre-mRNA splicing, and DNA methylation (Bannister et al., 2005; Vakoc et al., 2006; Kolasinska-Zwierz et al., 2009; Dhayalan et al., 2010; Venkatesh et al., 2012; Li et al., 2013; Pfister et al., 2014; Yuan et al., 2017). Furthermore, SETD2 also has non-histone substrates such as tubulin, actin, and STAT1 (Park et al., 2016; Chen K et al., 2017; Seervai et al., 2020). SETD2 is frequently mutated in cancers such as CCRCC (Clear Cell Renal Cell Carcinoma), and its deletion is embryonically lethal (Al Sarakbi et al., 2009; Duns et al., 2010; Hu et al., 2010; Zhu et al., 2014; Li et al., 2016a; Su et al., 2017; Chiang et al., 2018).

Although SETD2 is functionally important, the protein does not accumulate in human cells. Previously, we and others have shown that the robust degradation of SETD2 is brought about by the ubiquitin-proteasome system (UPS) (Zhu et al., 2017; Bhattacharya and Workman, 2020; Bhattacharya et al., 2021c). Notably, an extended N-terminal region is present in SETD2 that is absent in Set2, its yeast homolog. Interestingly, a protein-coding splice variant of SETD2 lacks the extended N-terminus but contains the catalytic SET domain as per the ENSEMBL database. Previously, we reported that this unique N-terminal region is rich in intrinsically disordered regions (IDRs) and facilitates SETD2’s degradation by the proteasome (Bhattacharya et al., 2021c). Consequently, the removal of this region leads to SETD2 accumulation (Bhattacharya and Workman, 2020; Bhattacharya et al., 2021c). However, the possible consequences of the cellular accumulation of SETD2 are unknown.

Here we show that increased SETD2 levels result in the spurious deposition of the H3K36me3 mark that occurs in a non-canonical manner. This is accompanied by widespread transcription and alternative splicing aberrations as well as phenotypic changes.

## Materials and methods

### Plasmids

SETD2-HaloTag^®^ human ORF in pFN21A was procured from Promega. Deletion mutants of SETD2 were constructed by PCR (Phusion polymerase, NEB) using full-length SETD2 as a template and individual fragments were cloned. All constructs generated were confirmed by sequencing.

Cell line maintenance and drug treatment- HEK293T cells were procured from ATCC. Cells were maintained in DMEM supplemented with 10% FBS and 2 mM L-glutamine at 37°C with 5% CO_2_. Transfections were performed at cell confluency of 40% using Fugene HD (Promega) using a ratio of 1:4 of the plasmid (μg) to transfection reagent (μl).

### Microscopy

293T cells were plated onto glass coverslips in a 6-well plate. Cells were washed with 1x PBS and fixed in 4% paraformaldehyde for 20 min at 37°C. Cells were then washed three times with cold 1x PBS. All the images depicted in the same panel were captured using the same settings unless otherwise specified.

### MTT assay

The viability of cells was quantified by their ability to reduce MTT to formazan, which is colored. Briefly, a thousand cells were plated per well, MTT reagent (5 mg/ml in Dulbecco’s PBS), was added to the cells at 1/10 volume of the medium. Cells were then incubated at 37°C for 4 h. A twofold volume of MTT solubilization buffer (0.01 M HCl; 10% SDS) was added to the cells, followed by incubation in the dark at 37°C for 24 h. The absorbance was measured at 570 nm with a TecanM200 microplate reader. Cell viability was expressed as the number of viable cells for each day.

### Migration and invasion assay

Migration and invasion assays were performed using a Transwell system (8-μm pore size; Corning Inc., Corning, NY, United States). Briefly, 1×10^5^ cells in 200 μl DMEM without serum were seeded onto the upper chamber of the Transwell (12-well insert). DMEM supplemented with 20% FBS was added to the basal compartment to serve as a chemoattractant. The cells were allowed to migrate for 24 h, and then, the cells that remained on the top side of the chamber were gently scraped off using wet cotton swabs. The cells that had migrated to the basal side were fixed in paraformaldehyde for 20 min at room temperature, stained with 0.2% crystal violet. Captured photographs were analyzed using ImageJ software.

For invasion assays, the chamber was coated with 100 μl of 1 mg/ml Matrigel (BD Biosciences, San Jose, CA, United States) and after overnight incubation, 2 × 10^5^ cells in DMEM without serum were plated on the apical side of the chamber. The other procedures were carried out in the same manner as the migration assay after 48 h of incubation. Matrigel was diluted 1:8 times for this assay.

### Isolation of total RNA and PCR

Total RNA was extracted from cells as per the manufacturer’s (Qiagen) instructions. To degrade any possible DNA contamination, isolated RNA was treated with DNaseI (NEB) for 30 min at 72°C. RNA (2 μg) was subjected to reverse transcription using the QScript cDNA synthesis mix according to the manufacturer’s instructions. cDNAs were then amplified with the corresponding gene-specific primer sets. For RT-PCR, PCR was conducted for 24 cycles using the condition of 30 s at 94°C, 30 s at 60°C and 30 s at 72°C. The PCR products were analyzed on 1% agarose gels containing 0.5 μg/ml ethidium bromide. The sequence of oligos is in Supplementary Figure S4.

### Antibodies

H3K36me3 (CST, 4909S), H3K36me2 (Active Motif, 39255), H3K36me1 (Abcam, ab9048), H3 (CST, 9715S), Halo (Promega, G9211), SETD2 (Abclonal, A3194), β-actin (Abcam, ab8224).

### ChIP

Cells were cross-linked in 1% formaldehyde for 10 min, and then the reaction was quenched by incubation in 125 mM glycine for 5 min. After washing with cold 1x PBS three times, cells were harvested by scraping using a cell scraper and pelleted down by centrifugation. The cell pellet was resuspended in the swelling buffer (25 mM HEPES pH 8, 1.5 mM MgCl_2_, 10 mM KCl, 0.1% NP40, 1 mM DTT, protease inhibitor cocktail), kept on ice for 10 min, and then subjected to Dounce homogenization. The nuclear pellet was obtained by centrifugation and resuspended in the sonication buffer (50 mM HEPES pH 8, 140 mM NaCl, 1 mM EDTA, 1% Triton X 100, 0.1% Na-deoxycholate, 0.1% SDS, protease inhibitor cocktail), followed by sonication for 12 cycles (30% amplitude, 10 s on/60 s off) using a Branson Sonicator while keeping on ice. For spike-in normalization, the spike-in chromatin and antibody were added to the reaction as per the manufacturer’s recommendation (Active Motif). The chromatin was incubated with antibodies at 4°C overnight and then added to 30 μl of equilibrated protein G-Dyna beads (Thermo Fisher Scientific) for an additional 2 h with constant rotation. The beads were extensively washed, and bound DNA was eluted with elution buffer (50 mM Tris-HCl pH 8, 5 mM EDTA, 50 mM NaCl, 1% SDS) and reverse-crosslinked at 65°C overnight. DNAs were purified using the QIAquick PCR purification kit (Qiagen) after the treatment of proteinase K and RNase A.

### High throughput sequencing

Sequencing libraries were prepared using High Throughput Library Prep Kit (KAPA Biosystems) following the manufacturer’s instructions. The library was sequenced on an Illumina HiSeq platform with paired reads of 75 bp for RNA-seq and single reads of 50 bp for ChIP-seq.

### ChIP-seq analysis

Raw reads were demultiplexed into FASTQ format allowing up to one mismatch using Illumina bcl2fastq2 v2.18. Reads were aligned to the human genome (hg38) using Bowtie2 (version 2.3.4.1) with default parameters (Langmead and Salzberg, 2012). For samples with fly spike-in, reads were first mapped to the *Drosophila melanogaster* genome (dm6), and unmapped reads were then aligned to the human genome (hg38). Reads per million (RPM) normalized bigWig tracks were generated by extending reads to 150bp. For spike-in ChIP-seq data, we also generated spike-in normalized bigWig tracks (RPM normalization factor = 1E6/number of reads aligned to hg38, and spike-in normalization factor = 1E6/number of reads aligned to dm6).

### Peak calling

Epic2 (with options: −gn hg38 −fs 200 −fdr 0.05) was used to call wide peaks for H3K36me3 ChIP-seq data for FL, C, and CΔSRI (Stovner and Sætrom, 2019). Next, ChIPseeker was applied (with options: genomicAnnotationPriority = c(‘Intergenic', ‘5UTR′, ‘3UTR′, ‘Exon', ‘Intron')) to obtain the genomic feature distribution (Ensembl 96 release) under peaks (Yu et al., 2015).

Metagene Plots- 14533 Protein-coding genes (Ensembl 96 release) were selected with length ≥600bp and no other genes within -2 Kb TSS and +2 Kb TES regions. Metagene regions were from -2 Kb TSS to +2 Kb TES. In addition, 2 Kb upstream TSS and downstream TES regions are grouped into 100 bins (20bp per bin), respectively. The gene body region is grouped into 300 bins (at least 2bp per bin since the minimum gene length is 600bp). In total, each gene is grouped into 500 bins. The average normalized (RPM or spike-in) H3K36me3 signals in each bin were plotted using the R package EnrichedHeatmap (Gu et al., 2018).

H3K36me3 on exons and introns- Protein-coding genes were selected, and the longest transcript for each gene was chosen. Also, we removed any overlapping transcripts (ignore the strand). As a result, 15311 transcripts were used to calculate the H3K36me3 signal (RPM normalized) distribution on exons/introns. The average exon/intron signal is defined as the total H3K36me3 signals on all exons/introns divided by the total exon/intron length.

### RNA-seq analysis

Raw reads were demultiplexed into FASTQ format allowing up to one mismatch using Illumina bcl2fastq2 v2.18. Reads were aligned to the human genome (hg38 and Ensembl 96 gene models) using STAR (version STAR_2.6.1c) (Dobin et al., 2012). TPM expression values were generated using RSEM (version v1.3.0) [5]. edgeR (version 3.24.3 with R 3.5.2) was applied to perform differential expression analysis, using only protein-coding and lncRNA genes (Robinson et al., 2010). rMATs (version 4.0.2) was used to perform differential alternative splicing analysis with default parameters starting from FASTQ files (Shen et al., 2014). FDR cutoff of 0.05 was used to determine statistical significance.

## Results

### The H3K36me3 mark is enriched over the 3′end and exons of transcriptionally active genes

Several methyltransferases, such as ASH1L, NSD1 and 2, etc., can deposit the histone H3K36me1 and H3K36me2 marks in mammalian cells (Wagner and Carpenter, 2012; Husmann and Gozani, 2019). However, SETD2 is the only enzyme that performs H3K36 trimethylation *in vivo*. Previously we showed that SETD2 stabilization results in an increase in global H3K36me3 levels in a Pol II-independent manner (Bhattacharya and Workman, 2020; Bhattacharya et al., 2021c).

**FIGURE 1:**
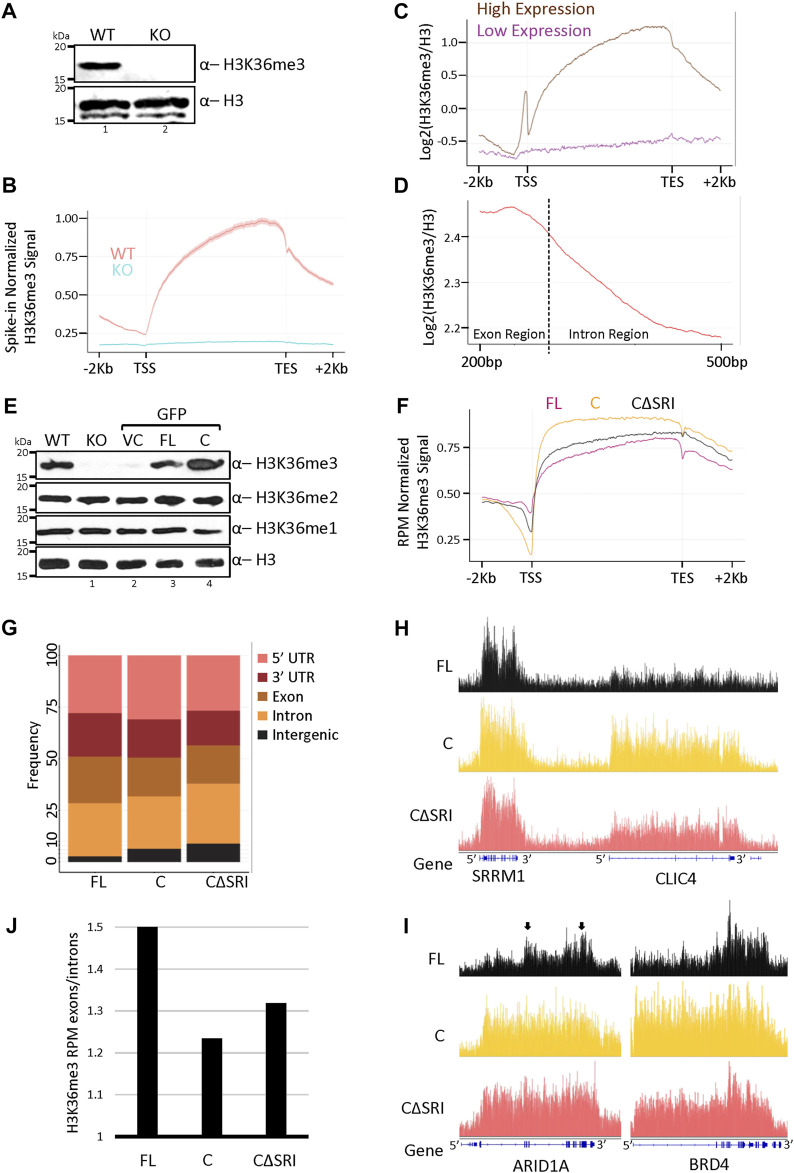
Increased SETD2 abundance leads to a non-canonical H3K36me3 deposition. **(A,E)** Western blot of whole-cell lysates probed with the depicted antibodies. setd2Δ 293T (KO) cells were transfected with GFP-vector control (VC), SETD2 full-length (FL), or SETD2 C **(C)** and lysates were prepared 72 h post-transfection. WT is wildtype. **(B–D,F)** Metagene plots depicting the normalized distribution of H3K36me3 in WT, KO (*setd2Δ* 293T), and KO cells expressing SETD2 constructs. **(G)** Histogram depicting the distribution of H3K36me3. The dotted line in **(D)** is representing the intron/exon junction. In our analysis, the average length for the exon was 326 base pairs (median was 139 base pairs), and the average length for the intron was around 4600 base pairs (median around 1080 base pairs). Hence, a shorter exon region length and longer intron region length is depicted in the H3K36me3 metagene plot with exon/intron junction regions. **(H,I)** Genome browser tracks depicting the peaks of H3K36me3. The blue solid blocks on the X-axis denote the exons. The arrows highlight the greater enrichment of H3K36me3 over exon-rich regions of ARID1A. **(J)** Bar graph showing the H3K36me3 enrichment over exons as compared to introns.

To understand the molecular changes occurring in cells upon SETD2 stabilization, we first wanted to examine the effect of increased SETD2 accumulation on the H3K36me3 distribution. For this, spike-in normalized H3K36me3 ChIP-Seq of wild-type (WT) and setd2Δ (knockout, KO) cells was performed first to understand the canonical distribution of this mark. In the KO cells, both the alleles of the endogenous SETD2 gene are disrupted and as expected, it lacks the H3K36me3 mark as can be seen in western blots (Figure 1A) (Hacker et al., 2016). Metagene analysis revealed a clear enrichment of H3K36me3 within the coding region of genes in the WT cells (Figure 1B). As expected, the H3K36me3 signal was not observed in the KO cells (Figure 1B). Moreover, the enrichment of H3K36me3 was skewed towards the 3’ end of genes (Figure 1B) as was reported previously (Barski et al., 2007). Additionally, a metagene analysis of H3 normalized H3K36me3 levels on protein-coding genes showed that this modification is greatly enriched at the highly expressed genes as compared to the low expressed ones (Figure 1C). This is consistent with the known association of this mark with transcriptionally active genes (Wagner and Carpenter, 2012; Carvalho et al., 2013). Furthermore, a closer inspection of the H3 normalized H3K36me3 distribution within the coding region revealed that H3K36me3 is more enriched over exons as compared to introns (Figure 1D).

### High levels of SETD2 result in a non-canonical H3K36me3 distribution

To test the effect of SETD2 stabilization on H3K36me3 levels and distribution, constructs were made to express GFP- or Halo-tagged full-length (SETD2 FL) and the C-terminal segment of SETD2 (1404-2564, SETD2 C) under the control of a CMV promoter. Recombinant expression of SETD2 has been previously used to investigate the function of the protein (Carvalho et al., 2014; Chen K et al., 2017; Zhu et al., 2017; Chiang et al., 2018; Zhang et al., 2020). The constructs were introduced in KO cells and 72 h post-transfection whole-cell lysates were prepared and analyzed by western blotting. Consistent with our previous report, the expression of SETD2 C in KO cells led to a marked increase in the H3K36me3 level as compared to WT cells, and the rescue with SETD2 FL (Figure 1E) (Bhattacharya and Workman, 2020). The other two H3K36 methyl marks, H3K36me1, and H3K36me2, largely remained unchanged (Figure 1E). The expression of the vector control (VC) did not alter the levels of H3K36 methylation as expected (Figure 1E). Further, microscopy of cells expressing GFP-tagged constructs and western blotting of lysates of cells expressing Halo-tagged proteins confirmed that the expression of SETD2 C is markedly higher than that of SETD2 FL as we have previously reported (Supplementary Figures S1A,B)^3^. Notably, the level of ectopically expressed SETD2 FL was comparable to that of endogenous SETD2 (Supplementary Figure S1C). The robust degradation of the full-length SETD2 protein by the proteasome likely accounts for this, even though a CMV promoter was used to drive the expression of the constructs.

Next, ChIP-Seq of H3K36me3 was performed post introduction of GFP-SETD2 constructs in the KO cells, and the H3K36me3 distribution was compared. A SETD2 C mutant that lacks the Set2-Rpb1 Interaction (SRI) domain (SETD2 CΔSRI)and hence, cannot interact with Pol II was also included. Initially, a comparison of the H3K36me3 distribution of WT cells and KO cells expressing SETD2 FL was performed. The total number of H3K36me3 peaks between WT (9131) and SETD2 FL expressing cells (11422) were comparable (Supplementary Figure S2A). It has to be considered though that H3K36me3 peaks are very broad and that may result in some discrepancies in peak-calling. Nevertheless, approximately 92% of H3K36me3 peaks in WT cells overlapped with those in SETD2 FL. Moreover, the overall distribution of H3K36me3 was very similar in WT and SETD2 FL-expressing KO cells (Supplementary Figure S2B). Interestingly, upon the rescue of the KO cells with the SETD2 C construct, the H3K36me3 mark continued to be enriched within the gene bodies (Figure 1F). Notably, this was true even upon rescue with the mutant that lacks RNA Pol II interaction (SETD2 CΔSRI). This is consistent with the reports in yeast that suggests that the association with Pol II is required for Set2’s activation and not necessarily its chromatin recruitment (see Discussion). However, a closer inspection of the H3K36me3 distribution revealed that it is skewed towards the 5′ end of the genes in cells rescued with SETD2 C as compared to SETD2 FL (Figure 1F). Also, consistent with the global increase in the H3K36me3 signal observed in western blots, the expression of SETD2 C and CΔSRI resulted in a markedly higher number of H3K36me3 peaks- 24884 and 17817, respectively (Supplementary Figure S2A). Furthermore, a global analysis of the peaks revealed that there is a higher proportion of H3K36me3 at the intergenic regions post-rescue with SETD2 C as compared to SETD2 FL (Figure 1G). Besides, there was an aberrant deposition of H3K36me3 on numerous genes. For example, the genome browser tracks of the CLIC4 gene reveal a higher level of H3K36me3 in SETD2 C and CΔSRI rescued cells as compared to SETD2 FL (Figure 1H). Likewise, the 5′ skewing of H3K36me3 observed in the metagene analysis of cells expressing SETD2 C and CΔSRI was readily apparent in genes such as ARID1A and BRD4 (Figure 1I). Moreover, in SETD2 FL expressing cells two regions that are exon-rich in the ARID1A gene (marked by arrows) have higher enrichment of H3K36me3 as compared to intronic regions (Figure 1I). This pattern is not as distinct in C and CΔSRI expressing cells. Indeed, a global analysis revealed that the ratio of average exon signal divided by the average intron signal was lower in SETD2 C rescued cells as compared to cells rescued with SETD2 FL (Figure 1J). This suggests that upon the increased accumulation of SETD2, the relative enrichment of H3K36me3 over exons decreases.

Thus, the absence of the N-terminal region results in greater accumulation of SETD2 and results in global changes in the canonical H3K36me3 distribution pattern.

### Excessive SETD2 accumulation results in changes in transcription

**FIGURE 2:**
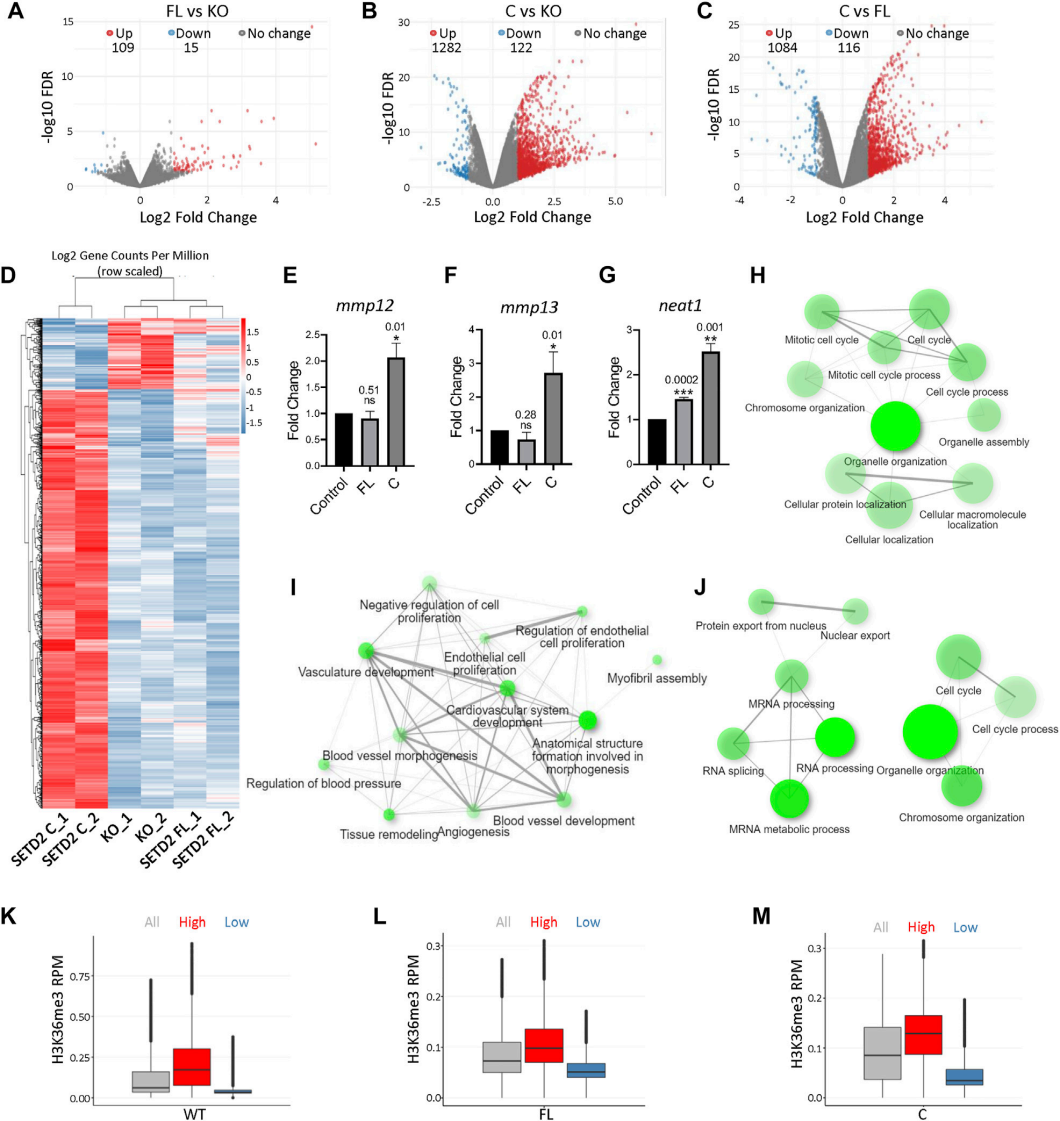
Removal of the SETD2 N-terminus leads to changes in gene expression. **(A–C)** Volcano plot showing the expression changes occurring upon expression of SETD2 FL and C in KO cells. Each dot represents a gene. RNA was isolated 72 h post-transfection. **(D)** Heat map showing differentially expressed genes upon the expression of SETD2 C and FL in KO cells. **(E–G)** RNA was isolated from KO cells 72 h post-transfection with GFP- control, SETD2 full-length (FL), or SETD2 C (C). Specific primers were designed to detect the indicated genes and quantitative PCR was performed. For each sample *n* = 3 independent biological samples were examined in the same sequencing run. Data are presented as mean values with a Standard Error of Mean. An unpaired *t*-test (two-tailed) was performed. *p*-value <0.05 was considered significant. *p*-values are depicted on the top of the respective graphs. GAPDH was used for normalization. **(H–J)** GO-term analysis of genes upregulated upon expression of SETD2 FL and SETD2 C in KO cells using ShinyGO (http://bioinformatics.sdstate.edu/go/). **(K–M)** Box plot showing the H3K36me3 and expression level of genes. The expression cutoffs are 0 < TPM≤2 and TPM≥4 to define low- and high-expressed protein-coding genes, respectively, in FL and C samples; and 0 < TPM≤0.1 and TPM≥0.5 to define low- and high-expressed lncRNA genes, respectively, in FL and C samples. In the boxplots, the black line inside the box shows the median. The box bottom and top border correspond to 25th and 75th percentiles (Q1 and Q3 respectively). The whiskers represent ranges from Q1—1.5 * IQR to Q3 + 1.5 * IQR where IQR stands for interquartile range (Q3—Q1). Data points outside the whiskers could be outliers and are marked as black dots.

To understand the effect of SETD2 accumulation on the transcriptome of cells, RNA-Seq was performed. The rescue of KO cells with SETD2 FL resulted in the differential expression of 124 genes [Fold change (FC) ≥2, false discovery rate (FDR) ≤0.05] (Figure 2A; Supplementary Data S1). Strikingly, SETD2 C expression in KO cells resulted in a much more pronounced effect with 1282 upregulated and 122 downregulated genes (Figure 2B; Supplementary Data S1). Additionally, a comparison of SETD2 C versus SETD2 FL-expressing KO cells revealed that a total of 1200 genes exhibited significant differential expression with the majority of them (1084) upregulated (Figures 2C,D; Supplementary Data S1). The higher expression of some of the genes observed in the RNA-Seq data was validated by performing qPCR (Figures 2E–G). The mRNA levels of SETD2 FL and SETD2 C were not significantly different in our RNA-seq data, suggesting that the differences observed are not due to the differences in the transcript abundance of the two SETD2 variants.

To understand the category of genes that are differentially expressed upon SETD2 expression, a Gene Ontology (GO) term analysis was performed. This revealed that the genes upregulated upon SETD2 C expression in KO cells were enriched in pathways related to the cell cycle (Figure 2H). By contrast, the genes that were upregulated upon SETD2 FL expression in KO cells were enriched in negative regulation of cell proliferation (Figure 2I). This is consistent with our previous report where SETD2 FL expression resulted in a decreased cell proliferation (Bhattacharya and Workman, 2020). The genes differentially expressed upon the comparison of SETD2 C and SETD2 FL datasets also revealed enrichment in cell cycle and mRNA processing-related pathways (Figure 2J). Furthermore, a closer inspection of the RNA-Seq data revealed that around 40% of genes upregulated upon SETD2 C expression as compared to SETD2 FL are long non-coding RNAs (lncRNA) (Supplementary Data S1).

Next, we looked for possible correlations between our RNA-seq and H3K36me3 ChIP-seq data. Analysis of H3K36me3 peaks in WT cells revealed that this mark is enriched on highly expressed genes (Figure 2K). In both SETD2 FL and C rescued cells, the H3K36me3 mark resembled the pattern found in WT cells with greater enrichment observed on highly expressed genes (Figures 2L,M).

Collectively, our data suggest that the removal of the N-terminal region of SETD2 resulted in high levels of H3K36me3 that positively correlated with increased gene expression.

### Deletion of the N-terminal region of SETD2 leads to changes in alternative splicing

**FIGURE 3:**
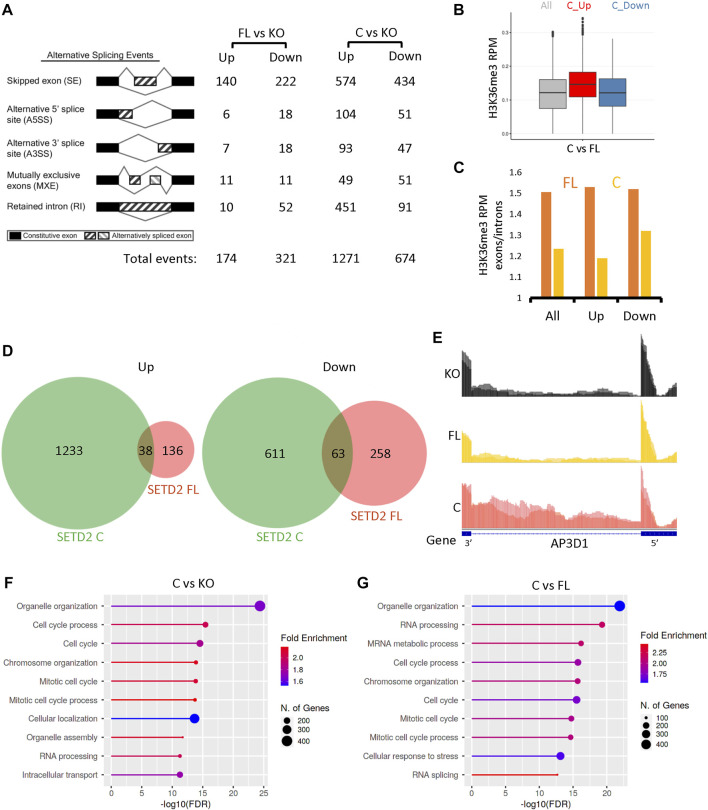
SETD2 accumulation leads to alternative splicing defects. **(A)** RMATS analysis depicting the differential AS events in SETD2 FL and SETD2 C expressing KO cells. The cartoon was taken from http://rnaseq-mats.sourceforge.net/. **(B)** A box plot showing the H3K36me3 level of genes that show differential alternative splicing upon SETD2 C expression as compared to SETD2 FL. In the boxplots, the black line inside the box shows the median. The box bottom and top border correspond to 25th and 75th percentiles (Q1 and Q3 respectively). The whiskers represent ranges from Q1—1.5 * IQR to Q3 + 1.5 * IQR where IQR stands for interquartile range (Q3—Q1). Data points outside the whiskers could be outliers and are marked as black dots. **(C)** A bar graph showing the H3K36me3 enrichment over exons as compared to introns of genes that show an increase (Up) or decrease (Down) in splicing events in C as compared to FL. “All” is the overall enrichment of all genes. For this analysis, for each protein-coding gene, the longest transcript was selected and the transcripts that overlapped with each other were removed (Ensembl 96 annotation). As a result, 15311 transcripts were used in the subsequent downstream analysis. Next, for each selected transcript, the mean H3K36me3 signal values in the exon and intron region were extracted and computed. An enrichment score was calculated as the ratio of the mean exon signal divided by the mean intron signal. **(D)** Venn diagrams showing the overlap of differential AS events resulting from the expression of SETD2 FL and SETD2 C in KO cells. **(E)** Genome browser tracks depicting the reads from RNA-Seq data. The blue solid blocks on the X-axis denote the exons. **(F,G)** GO-term analysis of differential AS events upon expression of SETD2 FL and SETD2 C in KO cells using ShinyGO (http://bioinformatics.sdstate.edu/go/).

Previously, we and others have shown that SETD2 is a regulator of alternative splicing (AS) of RNA (Luco et al., 2010; Chen et al., 2019; Bhattacharya et al., 2021a; Bhattacharya et al., 2021b). Hence, we looked for possible differences in AS in our data sets. RMATS analysis revealed changes in AS upon expression of SETD2 FL and SETD2 C in KO cells (Figure 3A; Supplementary Data S2). Similar to what was observed upon performing differential gene expression analysis, expression of SETD2C resulted in a much more pronounced effect on AS with a total of 1945 differential events (FDR ≤0.05) (Figure 3A; Supplementary Data S2). Out of these, 1271 events were increased and 674 were decreased upon SETD2 C expression (Figure 3A; Supplementary Data S2). The changes belonged to several kinds of splicing events, such as skipped exon, alternative 5′ splice site, alternative 3’ splice site, mutually exclusive exons, and retained intron. Most changes belonged to skipped exon and retained intron events (Figure 3A; Supplementary Data S2).

Next, we analyzed the H3K36me3 exon enrichment of the genes showing differential AS events. The H3K36me3 levels were found to be higher at the genes that showed increased splicing in SETD2 C versus SETD2 FL (Figure 3B). Furthermore, an anticorrelation of exon enrichment score was observed with splicing event upon SETD2 C expression, meaning where splicing was down-regulated, a higher enrichment score was seen (Figure 3C). However, this negative correlation was not seen in FL expressing cells in which a constant exon enrichment value of around 1.5 was observed over the same genes (Figure 3C).

Furthermore, the analysis of the AS events also revealed that the events resulting from the expression of SETD2 C and SETD2 FL were largely non-overlapping (Figure 3D). Such difference can be visualized using the genome browser tracks of genes such as AP3D1 which shows the greater retention of intron 23 upon SETD2 C expression (Figure 3E). Notably, the GO-term analysis revealed that the differential AS events caused by SETD2 C expression in KO cells were enriched in pathways belonging to the cell cycle (Figure 3F). Similar to gene expression analysis, the differential AS events upon the comparison of SETD2 C and SETD2 FL datasets revealed enrichment in pathways belonging to cell cycle and RNA processing (Figure 3G).

To conclude, the removal of the N-terminal region of SETD2 resulted in changes in AS and the genomic distribution of H3K36me3.

### SETD2 stabilization results in phenotypic changes that correlate with the changes in the transcriptome

Analysis of the transcriptome of cells revealed that the expression of SETD2 C resulted in the differential expression and splicing of numerous genes associated with the regulation of the cell cycle. Hence, we next investigated whether SETD2 stabilization is associated with phenotypic changes.

**FIGURE 4:**
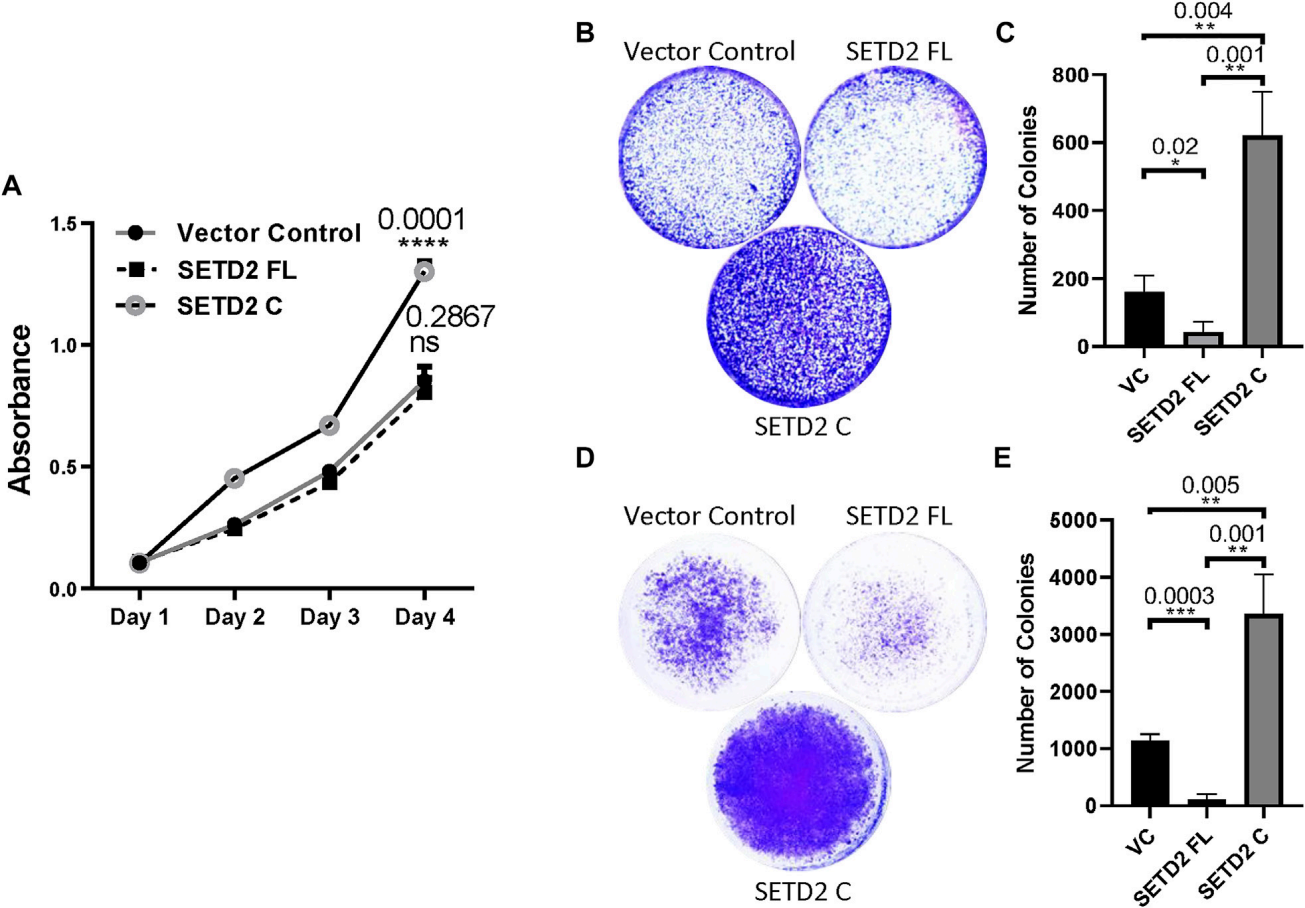
SETD2 accumulation results in cancer-related phenotypes. **(A)** A plot showing the results of the MTT assay to check cell proliferation. *n* = 3 independent biological samples examined in 3 independent experiments. ANOVA (2-way) was performed. *p*-value < 0.05 was considered significant. **(B–E)** Crystal violet-stained plates of invasion and migration assays were performed using KO cells expressing GFP-SETD2 FL and SETD2 C along with the associated quantification. *n* = 3 independent biological samples examined in 3 independent experiments. An unpaired *t*-test (two-tailed) was performed. *p*-value < 0.05 was considered significant. Data are presented as mean values with a Standard Error of Mean.

To this end, the effect on cell proliferation was tested by performing MTT [3-(4,5-dimethylthiazol-2-yl)-2,5-diphenyltetrazolium bromide] assay. It is a colorimetric assay for assessing the number of viable cells present by estimating the metabolic activity of cells. Consistent with our previous report, SETD2 C expression led to a significant increase in cell proliferation (Figure 4A) (Bhattacharya and Workman, 2020). Next, Transwell and Matrigel assays were performed to inspect the migration and invasion of cells, respectively. SETD2 C expression in KO cells resulted in their increased migration and invasion as can be seen by the pronounced crystal violet staining as compared to the vector control which suggests a greater number of cells (Figures 4B–E). The rescue of KO cells with SETD2 FL led to opposite effects with cells showing reduced invasion and migration, consistent with the tumor-suppressive role of SETD2 (Li et al., 2016b; González-Rodríguez et al., 2020; Hu et al., 2020) (Figures 4B–E).

Collectively, our data suggest that the removal of the SETD2 N-terminal region results in widespread molecular changes in mammalian cells and is associated with phenotypic abnormalities.

## Discussion

Although the role of the proteasome in regulating the stability of SETD2 is evident, it is not clear why the cellular half-life of SETD2 is so tightly regulated. This work follows up on our previous reports in illustrating the importance of the poorly characterized N-terminal segment of SETD2 in governing its appropriate function. We discovered that SETD2 accumulation results in changes in the H3K36me3 distribution, the transcriptome, and the phenotype of cells. We cannot rule out that the rescue of the KO cells with SETD2 constructs under a non-native promoter might be responsible for some of the observed changes. Yet, the expression of FL and C results in markedly different effects, although both are under the control of the CMV promoter. Previously, we have shown that the CMV-driven expression of FL and C are comparable at transcript level (Bhattacharya and Workman, 2020). Hence, the difference in the protein abundance between FL and C is likely responsible for the majority of the observed changes.

We previously showed that when SETD2 accumulates in cells, its dependency on RNA Pol II is reduced for H3K36me3 deposition (Bhattacharya and Workman, 2020). Strikingly, the ChIP-Seq data of H3K36me3 suggests that association with RNA Pol II is not required for recruitment of SETD2 to chromatin as previously assumed, but rather for SETD2 enzymatic activity like we previously speculated (Bhattacharya and Workman, 2020). Studies performed in yeast have also shown that binding to Pol II is required for Set2’s enzymatic activation (Wang et al., 2015). Notably, the Set2ΔSRI ChIP profile shows enrichment at the coding sequence of genes that is skewed towards the 5′ end much like what we found for H3K36me3 when SETD2 CΔSRI is introduced in setd2Δ cells (Suzuki et al., 2016). Possibly, Set2/SETD2 can continue to engage with the transcription elongation complex even in the absence of its interaction with RNA Pol II. In the future, it will be interesting to ascertain how the H3K36me3 and Set2/SETD2 enrichment within the gene bodies occurs even without the Pol II association.

**FIGURE 5:**
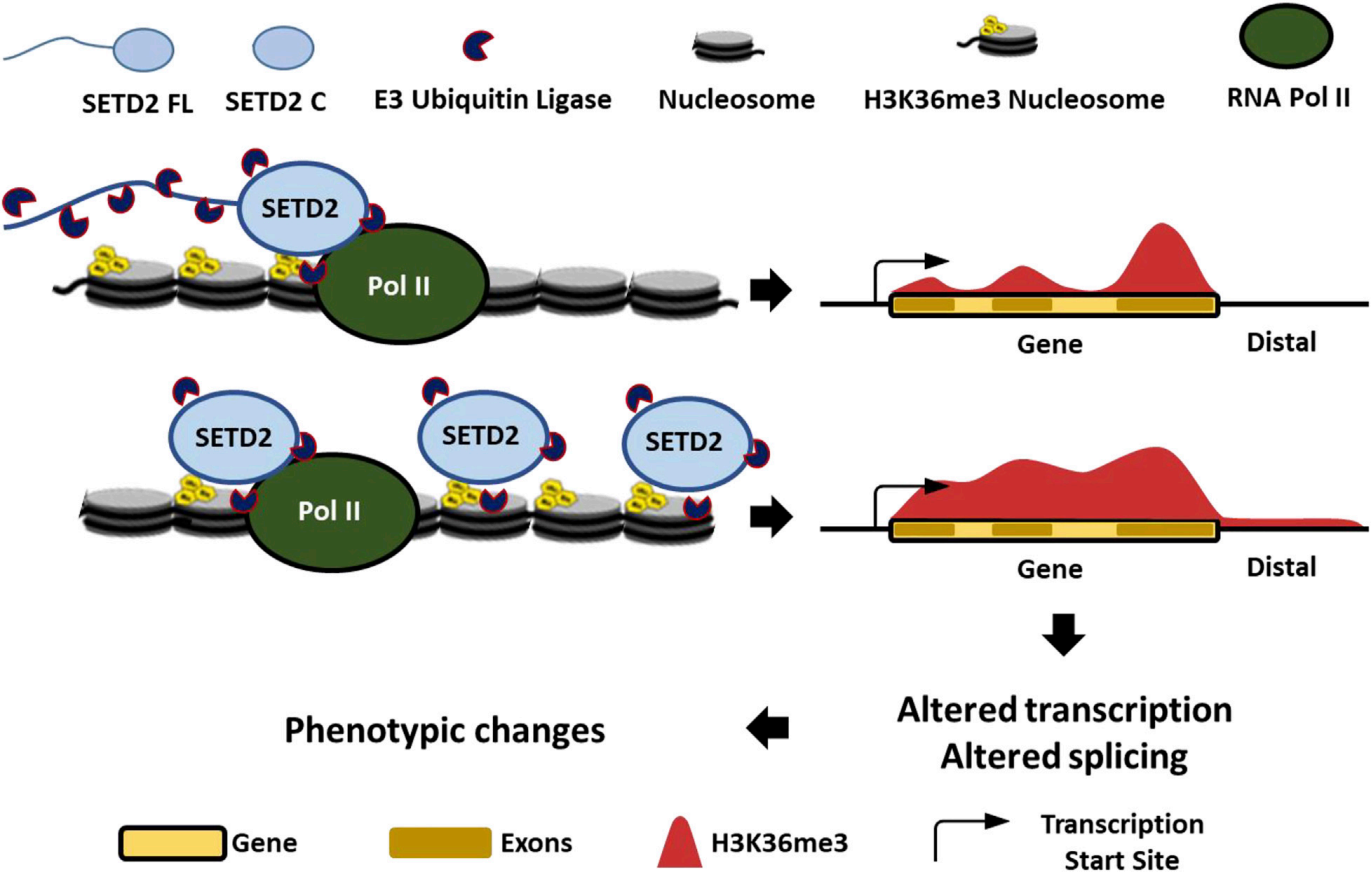
Proteolysis-mediated regulation of SETD2 levels is important to maintain its appropriate function.

Although H3K36me3 continued to be enriched within the gene bodies on expressing the stable variants of SETD2, it differed from the canonical distribution with reduced enrichment over exons and more presence in intergenic regions [Figure 5]. The disordered N-terminus region of SETD2 enables its robust degradation by the proteasome [Figure 5] (Bhattacharya et al., 2021c). This maintains SETD2’s dependency on Pol II-mediated activation for the deposition of the H3K36me3 mark. However, removal of the N-terminus region results in SETD2 accumulation that causes Pol II-independent deposition, as well as a non-canonical deposition of the H3K36me3 mark [Figure 5]. This non-canonical distribution of the H3K36me3 modification that is a known transcription activation mark, and a regulator of alternative splicing may result in the transcriptome changes that we observed. The list of aberrantly expressed genes includes NEAT1, MALAT1, MMP10, MMP12, and MMP13 that not only are functionally important but also have disease relevance (Vaalamo et al., 1998; West et al., 2014; Hirata et al., 2015; Chen T et al., 2017). Consistent with the upregulation of these genes, we saw increased migration and invasion of cells in our phenotypic assays [Figure 4]. Notably, a subset of genes that were downregulated in setd2Δ mouse embryonic stem cells (mESCs) belonged to the MAPK pathway (Zhang et al., 2014). Consistent with this, MAPK pathway genes such as the DUSP1, 2, and 5 were upregulated upon SETD2 accumulation (Supplementary Data S1). Furthermore, 52 genes from the ZNF family were upregulated upon SETD2 accumulation that are implicated in transcriptional regulation, signal transduction, and cell migration (Supplementary Data S1) (Cassandri et al., 2017). It is also striking that the transcript abundance of numerous lncRNAs was increased upon SETD2 C expression. SETD2 C interacts with the RNA-processing proteins the hnRNPs and also forms intranuclear phase-separated condensates (Bhattacharya et al., 2021a; Bhattacharya et al., 2021c). SETD2 has been shown to bind lncRNA like MALAT1 (Zhang et al., 2020). In the future, it will be interesting to investigate whether these properties of SETD2 C contribute to the transcript abundance changes that were observed.

The highly expressed genes upon SETD2 C expression also had higher H3K36me3 levels, consistent with the association of this mark with transcription activation. However, it is noteworthy that increased H3K36me3 levels did not always contribute to increased gene expression as we observed for the CLIC4 gene. Besides affecting the transcriptome, important epigenetic regulators like DNMT3a, MutSα, and MORF may be mistargeted by the aberrant H3K36me3 as we have suggested previously (Bhattacharya and Workman, 2020). Expression of the SETD2 shorter splice variant with transcript ID ENST00000638947.1 might lead to similar perturbations in the H3K36me3 profile to what was observed upon expression of SETD2 C. Also, the missense and truncation mutations found in SETD2 in cancers can potentially alter half-life, leading to aberrant H3K36me3 deposition (Supplementary Figure S3). SETD2 is a very important protein and the cell tightly regulates its accumulation as too much of it results in inadvertent consequences.

## Data Availability

The datasets presented in this study can be found in online repositories. The names of the repository/repositories and accession number(s) can be found below: Gene Expression Omnibus (GEO) database under the. accession number GSE147752. Original data underlying this manuscript can be accessed from the Stowers Original Data Repository at http://www.stowers.org/research/publications/libpb-1725.
