# Supplementary material for: Elevated levels of the methyltransferase SETD2 causes transcription and alternative splicing changes resulting in oncogenic phenotypes
Source: Front Cell Dev Biol. 2022 Aug 10;10:945668. doi: 10.3389/fcell.2022.945668 (PMC9399737; doi:10.3389/fcell.2022.945668)
Supplement: Supplementary file 1 [file DataSheet2.PDF]

Supplementary Information

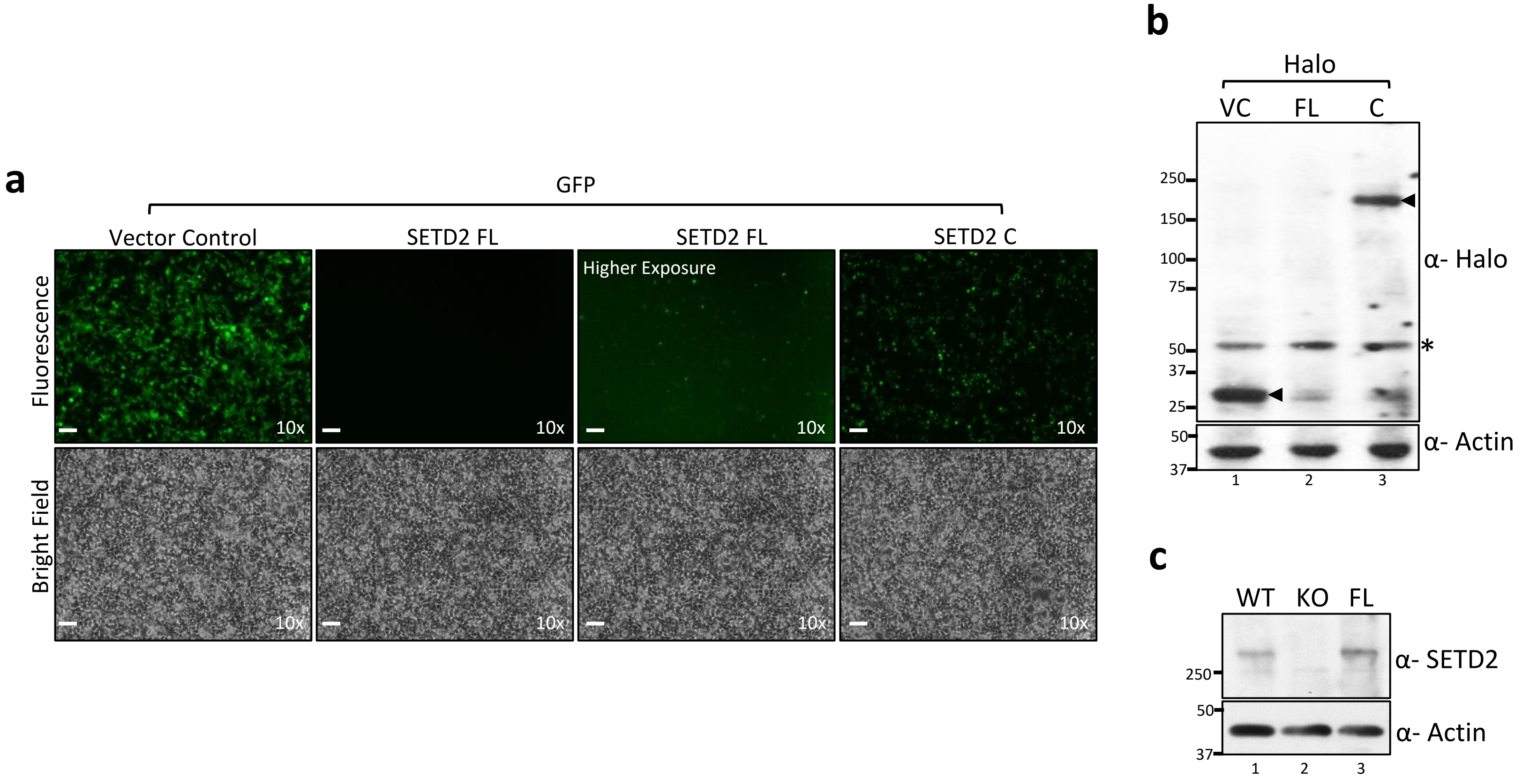

Figure S1: (a) Microscopy images showing the expression of GFP-SETD2 constructs in 293T cells. (b) Western blot of whole-cell lysates probed with the depicted antibodies. setd2Δ 293T (KO) cells were transfected with Halo-vector control (VC), SETD2 full-length (FL), or SETD2 C (C) and lysates were prepared 72 hrs post-transfection.* denotes non-specific band. The expected bands for the target are depicted by arrows. (c) Western blot of whole-cell lysates probed with the depicted antibodies. In FL, setd2Δ 293T (KO) cells were transfected with GFP-SETD2 full-length and lysates were prepared 72 hrs post-transfection. WT is Wild Type.

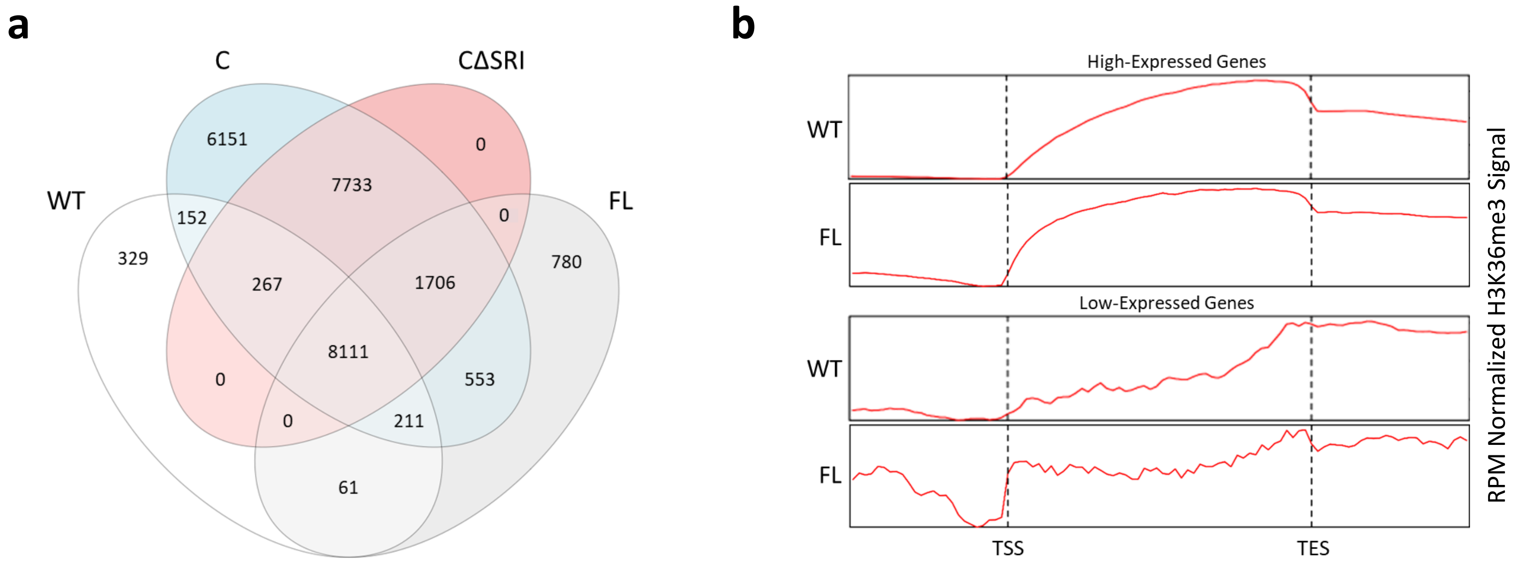

Figure S2: (a) Venn diagram showing the overlap of H3K36me3 peaks between different samples. (b) Metagene plots depicting the normalized distribution of H3K36me3 in WT and SETD2 FL-expressing KO cells.

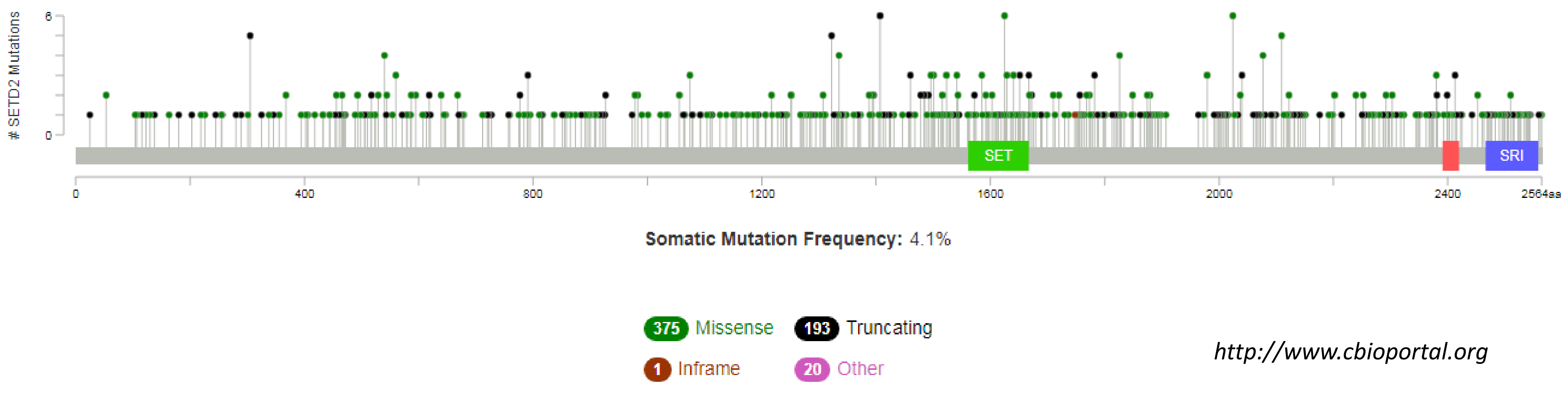

Figure S3: Cartoon depicting the location of various mutations found in SETD2 gene of cancer patients that may result in the generation of truncated forms of SETD2 with altered half-lives.

Oligo	Sequence (5’-3’)
MMP12_F	AACCAACGCTTGCCAAATCC
MMP12_R	TTTCCCACGGTAGTGACAGC
MMP13_F	CATGAGTTCGGCCACTCCTT
MMP13_R	CCTCGGAGACTGGTAATGGC
NEAT1_F	TCGACCCCTATCACATTGCC
NEAT1_R	CTCAGCAGTCTGCAGACC
GAPDH _F	TTCGACAGTCAGCCGCATCTTCTT
GAPDH _R	CAGGCGCCCAATACGACCAAATC

Figure S4: Sequence of oligos used to perform qPCR.
